# Mortality among Former Love Canal Residents

**DOI:** 10.1289/ehp.11350

**Published:** 2008-10-01

**Authors:** Lenore J. Gensburg, Cristian Pantea, Edward Fitzgerald, Alice Stark, Syni-An Hwang, Nancy Kim

**Affiliations:** 1 University at Albany, State University of New York, Albany, New York, USA; 2 New York State Department of Health, Troy, New York, USA

**Keywords:** community health, exposure assessment, hazardous waste sites, Love Canal, mortality

## Abstract

**Background:**

The Love Canal is a rectangular 16-acre, 10-ft deep chemical waste landfill situated in a residential neighborhood in Niagara Falls, New York. This seriously contaminated site first came to public attention in 1978. No studies have examined mortality in the former residents of the Love Canal neighborhood (LC).

**Objective:**

The aim of this study was to describe the mortality experience of the former LC residents from the years 1979–1996.

**Methods:**

From 1978 to 1982, 6,181 former LC residents were interviewed. In 1996, 725 deaths from 1979–1996 were identified in this cohort, using state and national registries. We compared mortality rates with those of New York State (NYS) and Niagara County. Survival analysis examined risks by potential exposure to the landfill.

**Results:**

We were unable to demonstrate differences in all-cause mortality for either comparison population for 1979 1996. Relative to NYS, the standardized mortality ratio (SMR) was elevated [SMR = 1.39; 95% confidence interval (CI), 1.16–1.66] for death from acute myocardial infarction (AMI), but not relative to Niagara County. Death from external causes of injury was also elevated relative to both NYS and Niagara County, especially among women (SMR = 1.95; 95% CI, 1.25 2.90).

**Conclusions:**

The role of exposure to the landfill in explaining these excess risks is not clear given limitations such as multiple comparisons, a qualitative exposure assessment, an incomplete cohort, and no data on deaths prior to 1978. Lack of elevation for AMI when compared with Niagara County but not NYS suggests possible regional differences. However, direct cardiotoxic or neurotoxic effects from landfill chemicals or indirect effects mediated by psychological stress cannot be ruled out. Revisiting the cohort in the future could reveal patterns that are not yet apparent.

The Love Canal is a rectangular 16-acre, 10-ft deep landfill centered in a residential neighborhood in northwestern New York State (NYS). The trench was originally dug in 1894 by William T. Love to connect the upper and lower Niagara Rivers, thereby providing cheap hydroelectric power. The landfill was one of the most seriously contaminated hazardous waste sites in the United States, containing approximately 21,800 tons of at least 200 different chemicals disposed by Hooker Chemical and Plastics Corporation from 1942 to 1953 [NYS Department of Health (NYSDOH) 1981]. According to company records, these chemicals were predominantly hexachlorocyclohexanes (e.g., lindane); benzylchlorides; organic sulfur compounds (e.g., lauryl mercaptans); chloro-benzenes; and sodium sulfide/sulfhydrates.

Contamination of homes adjacent to the landfill became apparent in 1978, with the potentially exposed population including several hundred residents within one block of the landfill and almost 3,000 residents within approximately four blocks (NYSDOH 1981). Environmental sampling, begun in the late 1970s, focused on indoor air, particularly in the basements and living spaces of homes closest to the landfill. Subsequent sampling included soil, sediments, water, leachate, and some biota. Possible migration routes, such as storm sewers and historic swales, were also examined. Excavation of the major swale found no evidence of migration along its bottom, but scattered, low-level contamination of the fill material suggested that chemically contaminated soils were used to fill the swales (Kim et al. 1982).

By 1980, several state and federal emergency declarations led to an emergency appropriation that helped purchase residences in the larger neighborhood surrounding the landfill, known as the Emergency Declaration Area (EDA) (Figure 1). This man-made disaster also prompted the passage of the Comprehensive Environmental Response, Compensation and Liability Act (CERCLA) by the U.S. Congress in 1980 (CERCLA 1980). This legislation authorized federal funding for Superfund remedial activities at hazardous waste sites nationwide.

In response to this situation, a number of health studies of the Love Canal neighborhood (LC) residents were conducted by the NYSDOH, the U.S. Environmental Protection Agency (EPA), and independent researchers. These studies examined blood counts and liver function tests (NYSDOH 1981), blood level of semivolatiles (Bristol et al. 1982), cytogenetic abnormalities and sister chromatid exchange (Heath et al. 1984; Picciano 1980), nerve conduction velocity (Barron 1982), rates of drug metabolism (Cuddy et al. 1984), cancer incidence (Janerich et al. 1981), low birth weight (Goldman et al. 1985; Vianna and Polan 1984), congenital malformations (Goldman et al. 1985; Paigen 1982), children’s growth rates (Paigen et al. 1987), and problems in childhood development (Paigen et al. 1985). The results of these studies were largely equivocal or contradictory, and none of the follow-up periods extended beyond 1982.

Concerns about long-term health effects due to residential exposure to the landfill prompted more recent research. In 1996, the NYSDOH began a series of studies to describe the health status of the former residents and their children through 1996. In 1998, an expert advisory committee was convened to provide advice and guidance. A year later, three former LC residents were added to the committee to provide community input. The objective of this study was to describe the findings for overall and cause-specific mortality by *a*) characterizing the mortality experience of the cohort from 1978 through 1996 compared with NYS [exclusive of New York City (NYC)] and Niagara County, and *b*) modeling mortality with regard to measures of potential exposure to chemicals from the landfill.

## Materials and Methods

### Study area and population

This follow-up health study cohort is based on the cohort that was identified and interviewed by the NYSDOH from 1978 to 1982. The 6,181 former residents included in the present study lived in the LC EDA any time between 1940 and June 1978, and were interviewed in 1978–1982 or, if < 18 years of age, one or both parents were interviewed.

Although Hooker Chemical did not begin using the trench to dump chemical waste until 1942, there was anecdotal evidence that chemical and municipal wastes were deposited there before 1942 (State of New York 1978). Because only 2.6% of the cohort lived in the EDA prior to 1940 and given that there is no clear date when waste was first deposited, 1940 was chosen as the year to begin exposure assessment. The date of entry into the study was the interview date; children were assigned the interview date of their parent.

By consulting City of Niagara Falls directories from the years 1940–1980 and using field staff to physically locate homes in 1978, we determined that there were 814 single-family homes in the EDA. Using information from the interviews, we found that of these homes, 776 (95%) were occupied by at least one member of the cohort sometime between 1940 and 1978, and 575 (74%) of the 776 homes were occupied by one or more members of the cohort for at least 75% of the time. A large portion of the EDA to the west of the landfill contained, sequentially, two public housing projects: Griffin Manor, which was torn down in the 1960s, and the LaSalle Development. Neither the number of apartments nor who resided in these projects is known; real property information is not available by apartment. The NYSDOH attempted to interview all residents living in the LaSalle project in 1978 by going door-to-door and setting up tables in the lobbies of the buildings, but the success rate of this attempt to include residents of the project is unknown. This interviewing process yielded 1,315 members of the cohort (21.3%) who resided in at least one of these rental units.

### Comparison populations

We chose New York State as a reference population because it was sufficiently large to provide stable death rates by year, age group, and sex (U.S. Census Bureau, Washington, DC). The five boroughs of NYC were excluded because their greater ethnic diversity would introduce potential confounding that could not be adjusted for in the analyses. Niagara County provided a comparison population very similar to the LC cohort demographically, while mitigating any potential regional differences in identifying the primary cause of death. Niagara County also allowed an attempt to control for possible local environmental sources of chemicals other than the landfill itself.

### Tracing of the cohort

We traced the 6,181 members of the cohort beginning in 1996 extending back to the date of their interview (1978–1982) to determine their current vital status and, if deceased, the date of death. The names of all females were first submitted to the NYS Vital Records (NYSVR) to be matched to the marriage registry for possible name changes. All names (e.g., birth, marriage) of both male and female members of the cohort were then matched to the Social Security Death Index database (ancestory.com 2009). The names of those not known to be dead were searched using NYS Department of Motor Vehicles (Albany, NY) files, Internet telephone directories, the U.S. Post Office Address Correction Service (U.S. Postal Service, Washington, DC), and the NYSVR Death Registry (NYSDOH Vital Records Bureau, Albany, NY). As a last resort, we contacted family members or former neighbors.

### Exposure assessment

In addition to comparisons with NYS and Niagara County, we conducted internal comparisons among members of the cohort using the potential for exposure of each resident to the landfill. We created an exposure matrix after a comprehensive review of files from the historical records (e.g., documented use of the landfill, odor complaints), environmental sampling data, and numerous interpretive reports. The matrix focused on location and time of residence plus three additional exposure-related variables: childhood exposure, attending the 99th Street School, and living in a residence on an environmental “hot spot” or historic swale.

**Figure 1:**
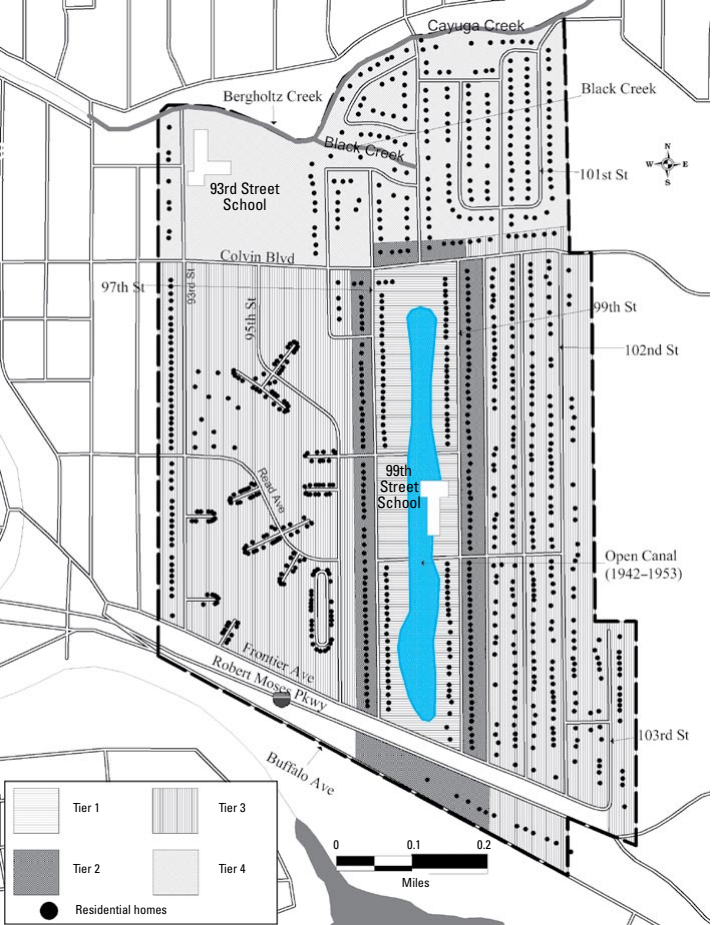
Emergency declaration area.

Location was defined by dividing the EDA, respectively, into four areas, or tiers: tiers 1 and 2, respectively, were contiguous to or across the street from the landfill; tiers 3 and 4 were farther away (Figure 1). Two distinct time periods of potential chemical exposure were identified: 1942–1953 and 1954 until evacuation (1978 for tiers 1 and 2 and 1980 for tiers 3 and 4). The few homes in tiers 1 and 2 in the earlier period would have been the most highly affected; all other residences were relatively less affected. Contaminants may have entered yards and homes through air transport and deposition, surface water runoff, and shallow ground-water transport during this period, especially in tier 1 (NYSDOH 1981).

The closed period began in 1954 when the landfill was covered and construction of homes in the area immediately adjacent was begun. These homes were situated such that either their back yards were contiguous with or directly across the street from the covered landfill. Odor complaints were made to local officials as early as the late 1950s and continued through 1978. The indoor environmental sampling of homes began in 1978, and > 800 air samples from 400 houses were collected. For chlorobenzene and chlorotoluene, the highest levels of contamination were in homes nearest the landfill (NYSDOH 1981). Thus, the historic and environmental evidence suggested a potential for exposure from 1954 until evacuation.

Individual residential history was determined and classified by time period and tier. Because of colinearity problems in the regression, tiers 1 and 2 were combined, as were tiers 3 and 4. The resulting variables consisted of four categories of potential residential exposure: *a* ) open period, tiers 1 and 2; *b* ) open period, tiers 3 and 4; *c*) closed period, tiers 1 and 2; and *d*) closed period, tiers 3 and 4. Cumulative exposure consisted of the number of years each study participant lived in each of the four tier/ time categories. These exposure estimates were not mutually exclusive, as many cohort members fell into more than one of the categories.

Childhood exposure was dichotomously defined as additional potential for exposure among children. Anecdotal evidence suggested that teenaged boys swam in the water-filled trench during the years of active dumping; therefore, 13- to 18-year-old males were considered potentially exposed in childhood from 1942 to 1953. After 1954, children < 13 years of age who lived closest (tiers 1 and 2) played on the soil covering the landfill and were therefore also considered potentially exposed during childhood. A second dichotomous variable indicated whether the cohort member lived in a residence either built on one of the natural historic swales or where the 1978 sampling results indicated higher than expected levels of chemical contaminants in the soil. The third additional exposure variable was the number of years of attendance at the 99th Street School, which had been built directly adjacent to the landfill.

To assess the sensitivity of the results because of the exposure definition used, we modeled three additional exposure classifications. One consisted of the total number of years a study participant resided in the EDA, irrespective of time period, location, or age. The remaining two definitions were based on four variables using age (≤ 18 years and > 18 years) and tier: *a*) ≤ 18 years*,* tiers 1 or 2; *b*) ≤ 18 years, tiers 3 or 4; *c*) > 18 years, tiers 1 or 2; and *d*) > 18 years, tiers 3 or 4. One definition quantified cumulative exposure using the number of years of residence in each of these four age and location combinations; the other dichotomized the four variables as ever/ never. Because the latter definition used indicator variables, the analyses were performed on a subset of the cohort in which the resulting variables were mutually exclusive.

### Outcome assessment

To obtain cause of death, the names of cohort members who were known to have died in the study period were matched with the NYSVR Death Certificate Registry (NYSDCR) and, if they died out of state, with the National Death Index (NDI) of the National Center for Health Statistics (Hyattsville, MD). First and any known last names, sex, race and dates of birth were submitted to the NYSDCR and/or NDI, and the underlying cause of death was abstracted using the *International Classification of Diseases, Ninth Revision* (ICD-9; Department of Health and Human Services 1989).

The Centers for Disease Control and Prevention’s (CDC) Wide-Ranging Online Data for Epidemiologic Research (CDC-WONDER; CDC 2007), a county-level national mortality and population database, was the source of the comparison mortality data. The mortality database is derived from records of deaths reported by each state’s vital records departments and reports all deaths for ages = 1 year. Data were collected by sex and age group for each year from 1979 to 1996. The preassigned age groups used by CDC-WONDER are 1–4, 5–9, 10–14, 15–19, 20–24, 25–34, 35–44, 45–54, 55–64, 65–74, 75–84, and ≥ 85 years. Data from each year were then grouped for analysis purposes as follows: June, 1978–1981, 1982–1986, 1987–1991, and 1992–1996. Deaths that occurred in the last 6 months of 1978 were considered to have the same rates as 1979. Data were included for any three-digit category of the ICD-9 for which there was at least one event in the cohort.

### Potential confounders

To control for potential confounding of the association between mortality and exposure, variables were abstracted from the 1978–1982 interviews. We abstracted information such as sex, date of birth, race, occupational narratives, and a history of cigarette smoking and alcohol consumption. The latter two variables were coded as ever/never. Occupational histories included job titles, company names, and dates of employment. NYSDOH industrial hygienists reviewed this information to evaluate each job’s potential for exposure to LC indicator chemicals (LCICs) as high, medium, or low/none. LCICs included chemicals such as β-hexachloro cyclo hexane, 2-chloronaphthalene, and 1,2,4-trichlorobenzene, known to have been deposited into the landfill and used to assess habitability of the EDA after containment (NYSDOH 1988).

### Statistical analysis

#### External comparisons

We computed person-years for the LC cohort as the difference of the date of interview to the date of death, loss to follow-up, or end of the study period (31 December 1996). We used a midyear assignment for persons for which only the year of death or loss to follow-up was known. Rates for each year group, age group, and sex were calculated for both NYS and Niagara County using the three-digit ICD-9 codes, both individually and grouped by organ system. Annual interpolations of the U.S. Census (U.S. Census Bureau, Washington, DC) were used to provide population estimates. The resulting rates were then multiplied by the respective person-years of observation for the LC cohort to calculate expected numbers of cases. Point estimates for standardized mortality ratios (SMRs) were computed as the ratio of observed to expected cases, and 95% confidence intervals (CIs) based on the Poisson distribution were calculated without adjustment for multiple comparisons. These age-adjusted and time period–adjusted SMRs were also calculated separately by sex for both NYS and Niagara County. Adjustments for race were not necessary because the percentages of whites in LC, NYS, and Niagara County were similar (95%, 93%, and 94%, respectively).

#### Internal comparisons

We used survival analysis, specifically the Cox proportional hazards model (Allison 1995; Hosmer and Lemeshow 1999), to model the association between the potential environmental exposure risk factors and survival time among members of the LC cohort; we also calculated hazard ratios (HRs). In keeping with the exploratory nature of the analysis, the models include all relevant environmental exposures and confounders, regardless of the resulting *p*-values.

The analyses focused on six categories of underlying cause of death: all causes; neoplasms (ICD-9 codes 140–239); circulatory system diseases (ICD-9 codes 390–459); acute myocardial infarction (AMI), a subset of circulatory system diseases (ICD-9 codes 410); respiratory system diseases (ICD-9 codes 460–519); and external causes of injury and poisoning (ICD-9 codes E800–E999). We chose these categories because of the large numbers of deaths experienced by the cohort in these groups.

Details concerning the study methodology have been published previously (NYSDOH 2008).

## Results

The LC cohort consists of 6,181 men, women, and children, of which 5,241 (84.8%) were known to be alive in 1996 with a known address; 725 (11.7%) died sometime in the follow-up period; 13 (0.2%) were known to be alive in 1996 but their current address was unknown; and 47 (0.8%) were lost to follow-up between the date of the interview and 1996 (Table 1). The demographic characteristics of the cohort by tracing status are presented in Table 2. In general, those traced and not traced were similar except those traced were slightly older (median age of 29 vs. 22 years) and therefore lived in the EDA slightly longer (8.5 vs. 5.0 years). More significantly, those traced were more likely to have lived only in single-family homes (78% vs. 51%, respectively; *p* < 0.0001). For the traced cohort, the median amount of time from first residential exposure to the end of the follow-up was 32 years (data not shown).

### External comparisons

After excluding 155 persons lacking vital status information, the remaining 6,026 people contributed 97,926 person-years to the analyses. Of the 725 deaths observed during the study period, 701 had cause-specific information; the remaining 24 deaths were reported by relatives and the cause was unknown. The latter deaths were included in all-cause mortality but omitted from cause-specific analyses.

Table 3 displays SMRs for females and males separately and with the sexes combined, with NYS as the standard population. Data are presented for specific causes with ≥ 10 expected deaths or a combination of an SMR > 1.0 and expected deaths > 5 for males and females combined. We discuss data using Niagara County as the standard population when they differ from those for NYS. Niagara County data have been reported previously (NYSDOH 2008).

For all-cause mortality, the SMR was 1.04 (95% CI, 0.96–1.12); for females, SMR = 1.00 (95% CI, 0.89–1.12); and for males, SMR = 1.06 (95% CI, 0.96–1.17). Similar to NYS and Niagara County, circulatory system diseases were the most common cause of death among the LC cohort (308 deaths; 42.5% of total). The SMR for men and women combined was 1.01 (95% CI, 0.90–1.13); for women alone, SMR = 0.93 (95% CI, 0.78–1.11); and for men, SMR = 1.06 (95% CI, 0.92–1.23). Death from an AMI was the most common in this category and was consistently elevated for both men (SMR = 1.37; 95% CI, 1.08–1.71) and women (SMR =1.43; 95% CI, 1.06–1.89). Cerebrovascular disease deaths were elevated in men only (*n* = 20; SMR = 1.13; 95% CI, 0.69–1.75). When using Niagara County as the standard population, the only important difference was the null finding for AMI [SMR in men = 1.00 (95% CI, 0.79–1.24); SMR in women = 1.04 (95% CI, 0.77–1.38)].

The second most common cause of death category among both reference populations and among the LC cohort was neoplasms (189 deaths; 26.1% of total). SMRs for neoplasms were ≤ 1.00 for both sexes combined and for men and women separately. For cause-specific analyses, the only SMR > 1.00 among women was 1.11 (95% CI, 0.71–1.65) for digestive system neoplasms, and among men, lymphatic and hematologic neoplasms (SMR = 1.06; 95% CI, 0.53–1.90) and other and unspecified sites (SMR = 1.52; (95% CI, 0.81–2.60).

Unlike NYS or Niagara County, the third most common cause of death category in the LC cohort was external causes of injury and poisoning (62 deaths; 8.6%). The SMR was 1.41 (95% CI, 1.08–1.81) for both sexes combined. This excess risk was greater among women (SMR = 1.95; 95% CI, 1.25–2.90) compared with men (SMR = 1.20; 95% CI, 0.85–1.65). Women had elevated SMRs for suicides (SMR = 2.35; 95% CI, 0.76–5.48), motor vehicle accidents (SMR = 2.12; 95% CI, 1.02–3.89), and other types of accidents (SMR = 1.52; 95% CI, 0.56–3.31). Suicides (SMR = 1.52; 95% CI, 0.79–2.66) and other types of accidents (SMR = 1.33; 95% CI, 0.69–2.32) were also elevated for men.

### Internal comparisons

Of the 6,026 traced cohort members, 5,974 had known vital status and dates of residence in the EDA. Of these, 706 were deceased, 5,221 were alive through 1996, and 47 were lost to follow-up some time after their interview and before 31 December 1996. Analyses were performed on the subset of 3,796 adults with complete interview data (85.2% of those interviewed) to control for possible confounders such as smoking, alcohol consumption, and occupation. The full study cohort and subset of interviewees were similar with respect to sex, race, and residence in the open period (data not shown). By definition, the interviewees, who had to be at least 18 years old to participate, were older and had longer residencies in the closed period than the cohort as a whole. For brevity’s sake, we present only the models for adults with complete interview data. The results for the models based on the complete cohort were virtually identical with respect to the exposure variables of interest.

As shown in Table 4, the risk for all-cause mortality increased with age (HR = 1.10; 95% CI, 1.09–1.10) and was higher among males (HR = 1.65; (95% CI, 1.36–2.02) and smokers (HR = 1.66; 95% CI, 1.35–2.05). The only elevated HR for all-cause mortality among the exposure variables was for childhood exposure (HR = 1.14; 95% CI, 0.54–2.42), but the number of deaths was small (*n* = 9). Age and male sex were also positive associations with several specific causes of death. For AMI, sex was time dependent, requiring an interactive term to be added to the model. Risk of death from AMI among males was greatest at the beginning of the follow-up period (HR = 4.28; 95% CI, 1.79–10.21) and decreased over the 18 years of follow-up (HR = 0.91). Smoking was also positively associated with cause-specific mortality risk: HRs ranged from 1.34 (95% CI, 0.84–2.12) for deaths from AMI to 6.23 (95% CI, 2.15–18.02) for deaths from respiratory system disease.

The four residential exposure variables representing tier and time period showed little association with cause-specific mortality (Table 4) with the exception of the closed period, tiers 1 or 2 for deaths from AMI (SMR = 1.06; 95% CI, 1.01–1.13). This finding was also time dependent; as the follow-up period progressed, the risk decreased to 0.99. The small numbers of residents living on a hot spot or a historic swale had no deaths from respiratory disorders or external causes of injury. The HR associated with attendance at the 99th Street School was elevated only for external causes of injury (HR = 1.12; 95% CI, 0.94–1.32). Childhood exposure had elevated HRs for both deaths from neoplasms and AMI, but the CIs were very wide because of small numbers, and no deaths from respiratory disease were observed for this variable.

## Discussion

These analyses were exploratory. The results describe the mortality status of the LC cohort and suggest directions for future research. Thus, we analyzed the data in several ways using more than one definition of exposure. No single finding should be overemphasized; interpretable, coherent patterns of findings are more likely to indicate valid and meaningful associations. For example, emphasis should be given to similar results when compared with both external control groups, along with those that showed consistent associations. It is also important to exercise caution in that, given the large number of statistical comparisons made, the likelihood of committing a type 1 error is much greater than the nominal 5%. Finally, qualitatively, the width of the CI is very informative: extremely wide CIs indicate that the findings are imprecise.

In the present study we were unable to demonstrate a difference in all-cause mortality for the years 1979–1996 compared with either NYS (exclusive of NYC) or Niagara County; we also could not detect differences for most individual causes of death. The most notable exceptions were deaths from AMI and from external causes, using the NYS reference population. When Niagara County was used as the comparison, the number of deaths from external causes remained excessive, but the death rate from AMI was no longer elevated. Consequently, it is possible that the excess mortality from AMI among LC residents relative to NYS is due to regional differences in mortality rates or in cause of death coding.

Comparison with earlier LC studies is not possible because no other investigation focused on mortality as an end point. However, in a study of another Niagara Falls waste site, no excess in cancer mortality was detected in three surrounding census tracts from 1973 to 1982 (NYSDOH, unpublished data), a finding consistent with that observed in the present study. Some other hazardous waste site studies have reported elevated mortality from specific cancers (Najem and Greer 1985; Najem et al. 1983, 1985; Najem and Molteni 1983), but others have not (Baker et al. 1988; Budnick et al. 1984; Najem et al. 1984, 1994; Polednak and Janerich 1989). Dunne et al. (1990) reported negative findings in an Australian population. Similarly, in a study of a community in South Wales surrounding a landfill site, Fielder et al. (2000) found no excess in all-cause mortality, cancer mortality, or respiratory disease. This study population lived within 3 km of a site used for household, commercial, and industrial wastes, and, like the LC landfill, the residents complained about noxious odors emanating from the site.

Assuming the observed associations of living in the EDA, with mortality from AMI, motor vehicle accidents, and suicides representing a causal relationship, one may postulate two possible pathways: *a*) direct cardiotoxic or neurotoxic effects leading, through biological mechanisms, to heart disease or to psychologic or behavioral symptoms; and *b*) indirect stress-induced physiologic or psychologic reactions, including elevated blood pressure and/or injurious behavioral reactions.

Neurotoxic effects have been reported from occupational exposure to organic solvents, largely among industrial painters (Parkinson et al. 1990; Triebig et al. 2000). At a community level, there is evidence for neuropsychologic effects, including anxiety and depression, from exposure to trichloroethylene (associations that were strongest in the context of alcohol consumption) (Reif et al. 2003). Among farmers, similar effects were associated with organophosphate pesticides (Beseler and Stallones 2003; Stallones and Beseler 2002). In the studies of farmers, one correlate of the neuropsychologic symptoms was a tendency not to follow safety practices (Beseler and Stallones 2003), a pattern with implications for injury risks.

As for heart disease, oxidative chemical injury is thought to be important in atherogenesis, potentially implicating a wide range of chemicals (Ramos 1999). Exposure to carbon disulphide (Kristensen 1989; Lewis et al. 1999), methylmercury (Stern 2005), arsenic (Bunderson et al. 2004), and bis (2-chloro-ethoxy) methane (Dunnick et al. 2004) has been shown to cause atherogenesis or myocardial damage in human, *in vitro*, and/or animal studies. Additional evidence has come from research on the toxicology of fine airborne particulate matter, found to be associated with cardiovascular disease in epidemiologic studies (Nemmar et al. 2004).

The stressors at LC consisted of a series of events over months and years, starting with the first reports of chemical contamination and continuing through the responses of governmental agencies, different investigations, relocation, and its aftermath. Effects of stress in other communities near hazardous waste sites have included physiologic reactions that constitute risk factors for cardiovascular disease: elevated blood pressure, elevated levels of stress hormones and catecholamines (Baum and Fleming 1993), demoralization (Horowitz and Stefanko 1989), and depression and anxiety (Foulks and McLellen 1992). Research supports the notion that at least a segment of the population reacts to stress with increased drinking (Holahan et al. 2001; Sillaber and Henniger 2004) or smoking (Carvajal et al. 2000; Kouvonen et al. 2005; Todd 2004). Alcohol consumption is a risk factor for injury outcomes, including suicide and motor vehicle crash injuries, whereas smoking is a risk factor for myocardial infarction and several cancers (Ezzati et al. 2002).

There was a significant excess risk of AMI for residents of tiers 1 and 2 during the closed period from 1954 to 1978 (Table 4). This may be a chance finding due to multiple comparisons, but it is consistent with the results of the external analyses using NYS as the standard. Interestingly, this excess risk was time dependent for men, disappearing by the end of the follow-up period. This finding suggests that the elevation in the risk of death from AMI, if real, was the result of acute and not chronic exposures or stressors. Several established risk factors for mortality, such as age, smoking, and male sex, were significantly associated with increased overall and cause-specific mortality, lending confidence to the overall design.

The study has several notable strengths. The cohort is well defined, with known residential locations and dates. Residents at the time of the evacuations were included, as well as persons who lived at LC before 1978. Exposures of 6 months to 39 years (median 8.5 years) were included, representing almost all areas of the EDA. Ninety-six percent of the cohort was successfully traced, minimizing an additional potential source of selection bias. We used two different, complementary research designs. One compared the cohort as a whole to two different standard populations; the other modeled potential internal differences in outcome associated with different exposures to the landfill while controlling for potential confounders. Mortality data obtained from death certificates avoid recall biases commonly associated with self-reported data. Although misclassification of the underlying cause of death may have occurred, such errors should be nondifferential with respect to exposure, attenuating rather than exaggerating any observed associations. Lastly, the study was conducted almost two decades after the crisis, allowing an adequate latency period to study chronic disease mortality.

Correspondingly, the study has several important limitations. By definition, the cohort is limited to residents who participated in interviews conducted in 1978–1982; not all former residents were identified at that time. Consequently, deaths that occurred before 1978 were excluded, possibly biasing the results toward the null. Despite a total of nearly 100,000 person-years of follow-up, statistical power was low for many specific causes of death, especially in the internal analyses resulting in small numbers and imprecision. Thus, for the most part, analyses were limited to the organ system level. Similarly, the cohort is relatively young and may not yet be at elevated risk of many causes of death despite the median of 32 years from first residential exposure to the end of follow-up. In the exposure assessment we used data from a wide variety of sources; data were, of necessity, qualitative because environmental sampling data were unavailable before 1978. Thus, exposure misclassification may have occurred, obscuring possible associations. However, serum samples archived from 1978 were available for 373 persons in the cohort and are being analyzed for concentrations of selected LCICs. These data may help validate time and location of residence as exposure surrogates. Finally, mortality is a relatively crude indicator of the effect of environmental exposures. Future investigations will focus on cancer incidence and adverse reproductive outcomes, which may be more sensitive end points in this population.

## Conclusion

This study was conducted to help assess, for the first time, the long-term health effects of residence at LC, the site of one of the first and most seriously contaminated hazardous waste sites in the history of the United States. The results did not demonstrate an elevation of overall mortality in the LC cohort compared with Niagara County or NYS from 1979 to 1996. There was some evidence of higher than expected death rates from AMI compared with NYS and from external causes of injury, principally suicide and motor vehicle accidents, compared with both NYS and Niagara County. The finding of no elevation for AMI compared with Niagara County suggests possible regional differences. However, persons who lived in tiers 1 and 2 during the closed period (1954–1978) had a higher risk of death from AMI. The role of exposure to the LC landfill in explaining these excess risks is not clear given limitations such as multiple comparisons, a qualitative exposure assessment, an incomplete cohort, and no death data prior to 1978. However, either direct cardiotoxic and neurotoxic effects from landfill chemicals or indirect effects mediated by psychologic stress cannot be ruled out. Because many analyses were limited by small numbers of deaths and because the study population is still relatively young (median age < 50 years in 1996), revisiting the cohort in the future could reveal patterns that are not yet apparent.

## Correction

In the original article published online, the list of authors was incorrect. Syni-An Hwang has been included here.

## Figures and Tables

**Table 1:** Results of tracing the 6,181 members of the Love Canal cohort.

Tracing results	No. (%)
Known to be alive in 1996 and current address is known	5,241 (84.8)
Known to have died in the follow-up period 1978–1996	725 (11.7)
Known to be alive in 1996 but current address is unknown	13 (0.2)
Lost to follow-up sometime from the date of interview to 1996	47 (0.8)
No information available	155 (2.5)
Total	6,181

**Table 2:** Demographic characteristics [no. (%)] of the Love Canal cohort (*n* = 6,181).

Cohort characteristics	Traced	Not traced
Total	6,026	155
Race		
White	5,717 (95.2)	130 (85.0)
Black	239 (4.0)	19 (12.4)
Other	48 (0.8)	4 (2.6)
Sex		
Male	2,914 (48.4)	50 (32.7)
Female	3,112 (51.6)	103 (67.3)
Residence type		
Single-family homes only	4,699 (78.0)	79 (51.0)
Public housing only	747 (12.4)	65 (41.9)
Public and single family	580 (9.6)	11 (7.1)
Year of entry into study		
1978	3,069 (50.9)	97 (62.6)
1979	652 (10.8)	10 (6.4)
1980	676 (11.2)	17 (11.0)
1981	1,353 (22.5)	25 (16.1)
1982	276 (4.6)	6 (3.9)
Living in the EDA in 1978		
Yes	3,099 (51.4)	92 (59.4)
No	2,927 (48.6)	63 (40.6)

**Table 3:** SMR, year and age adjusted, for females and males separately and combined compared with NYS (exclusive of NYC).

	Females			Males			Combined		
Cause of death	Observed	SMR	95% CI	Observed	SMR	95% CI	SMR	95% CI
All causes	309	1.00	0.89–1.12	416	1.06	0.96–1.17	1.04	0.96–1.12
Infectious disease	a	0.43	0.05–1.54	11	1.27	0.63–2.26	0.97	0.52–1.66
Human immunodeficiency virus	0	—	—	7	1.36	0.55–2.81	1.04	0.45–2.31
Neoplasm	83	0.87	0.69–1.08	106	1.00	0.82–1.21	0.94	0.81–1.08
Digestive system	24	1.11	0.71–1.65	25	0.89	0.57–1.31	0.98	0.73–1.30
Respiratory system	21	0.99	0.61–1.52	36	0.97	0.68–1.34	0.98	0.74–1.27
Bone, connective tissue, skin	12	0.54^b^	0.28–0.95	—	—	—	0.71	0.42–1.12
Genitourinary tract	12	0.91	0.47–1.59	14	0.91	0.50–1.52	0.91	0.59–1.33
Other and unspecified site	5	0.67	0.22–1.55	13	1.52	0.81–2.60	1.12	0.66–1.77
Lymphatic and hematologic	8	0.99	0.43–1.95	11	1.06	0.53–1.90	1.03	0.62–1.61
Endocrine and metabolic disease	7	0.81	0.33–1.67	7	0.82	0.33–1.69	0.82	0.45–1.37
Other endocrine glands	7	0.99	0.40–2.04	6	0.90	0.33–1.97	0.95	0.50–1.62
Diseases of the circulatory system	125	0.93	0.78–1.11	183	1.06	0.92–1.23	1.01	0.90–1.13
AMI	49	1.43^b^	1.06–1.89	77	1.37^b^	1.08–1.71	1.39	1.16–1.66
Chronic ischemic heart disease	30	0.70	0.47–1.00^+^	51	0.90	0.67–1.18	0.81	0.65–1.01
Other form of heart disease	20	0.91	0.55–1.40	22	0.85	0.53–1.28	0.87	0.63–1.18
Cerebrovascular diseases	16	0.73	0.42–1.19	20	1.13	0.69–1.75	0.91	0.64–1.26
Diseases of the respiratory system	29	1.20	0.81–1.73	28	0.93	0.62–1.34	1.05	0.79–1.36
Pneumonia and influenza	8	0.89	0.38–1.75	7	0.69	0.28–1.42	0.78	0.44–1.29
Chronic obstructive pulmonary disease	18	1.48	0.88–2.34	16	0.99	0.56–1.60	1.20	0.83–1.67
Other respiratory system	a	0.90	0.11–3.25	5	1.78	0.58–4.16	1.39	0.56–2.87
Diseases of the digestive system	10	0.86	0.41–1.58	23	1.57	0.99–2.35	1.26	0.86–1.76
Other digestive system	5	0.76	0.25–1.77	15	1.45	0.81–2.39	1.18	0.72–1.82
External causes of injury and poisoning	24	1.95^b^	1.25–2.90	38	1.20	0.85–1.65	1.41	1.08–1.81
Other accidents/adverse effects	a	1.52	0.56–3.31	12	1.33	0.69–2.32	1.39	0.82–2.19
Motor vehicle accidents	10	2.12^b^	1.02–3.89	10	0.90	0.43–1.65	1.26	0.77–1.95
Suicide	a	2.35	0.76–5.48	12	1.52	0.79–2.66	1.70	0.99–2.72

1.00^+^ , slightly > 1.00.

a For confidentiality, observed numbers of cases < 5 are not reported.

b 95% CI does not include 1.

**Table 4:** Cox proportional hazards modeling for mortality [HRs (95% CIs)], interviewees only (*n* = 3,796).

Variable	All causes of death (*n* = 620)	Neoplasms (*n* = 172)	Circulatory system (*n* = 272)	AMI^a^ (*n* = 116)	Respiratory system (*n* = 49)	External causes of injury and poison (*n* = 42)
Open period, tier 1 or tier 2 (years)	0.98 (0.89–1.08)	0.86 (0.64–1.16)	1.02 (0.92–1.15)	1.01 (0.86–1.20)	1.13 (0.92–1.38)	0.89 (0.39–2.02)
Open period, tier 3 or tier 4 (years)	0.99 (0.97–1.01)	0.98 (0.94–1.02)	1.00 (0.97–1.03)	0.98 (0.94–1.03)	0.99 (0.93–1.07)	1.02 (0.94–1.12)
Closed period, tier 1 or tier 2 (years)	1.00 (0.98–1.01)	1.01 (0.98–1.03)	1.00 (0.98–1.02)	1.06^b^ (1.01–1.12)	0.98 (0.94–1.03)	0.97 (0.91–1.04)
Closed period, tier 3 or tier 4 (years)	1.00 (0.99–1.01)	1.00 (0.99–1.02)	1.00 (0.99–1.01)	1.02 (1.00^–^–1.03)	0.99 (0.96–1.02)	0.90^b^ (0.82–0.99)
Hot spot/swale (yes/no)	0.91 (0.50–1.66)	1.11 (0.41–3.02)	1.35 (0.63–2.89)	0.83 (0.20–3.38)	c	c
Childhood exposure (yes/no)	1.14 (0.54–2.42)	2.50 (0.72–8.70)	0.98 (0.13–7.54)	2.70 (0.33–21.12)	c (0.16–2.91)	0.67
Years attending 99th Street School	0.96 (0.85–1.08)	0.58 (0.33–1.04)	0.56 (0.24–1.29)	0.52 (0.15–1.74)	c (0.94–1.32)	1.12
Age (years)	1.10^b^ (1.09–1.10)	1.09^b^ (1.08–1.10)	1.12^b^ (1.10–1.13)	1.11^b^ (1.09–1.13)	1.12^b^ (1.09–1.15)	1.01 (0.98–1.04)
Sex (male)	1.65^b^ (1.36–2.02)	1.50^b^ (1.03–2.18)	1.84^b^ (1.35–2.49)	4.28^b^ (1.79–10.21)	1.24 (0.62–2.46)	1.72 (0.82–3.62)
Ever smoked (yes/no)	1.66^b^ (1.35–2.05)	1.63^b^ (1.10–2.44)	1.36^b^ (1.00^+^–1.84)	1.34 (0.84–2.12)	6.23^b^ (2.15–18.02)	2.25 (0.93–5.45)
Alcohol consumption (yes/no)	0.91 (0.76–1.08)	1.15 (0.81–1.63)	0.87 (0.67–1.13)	0.80 (0.54–1.19)	1.65 (0.82–3.28)	1.16 (0.52–2.58)
Potential occupational exposure to LCICs (yes/no)	1.00 (0.83–1.21)	1.01 (0.70–1.45)	1.24 (0.92–1.66)	1.33 (0.85–2.11)	0.50^b^ (0.25–0.97)	0.94 (0.45–1.95)
Interactions with survival time						
Closed period (tiers 1/2)				0.99 (0.98–1.00)		
Closed period (tiers 3/4)						1.01 (1.00^+^–1.02)
Sex				0.91 (0.85–0.99)		

1.00^+^ , slightly > 1.00; 1.00^−^ , slightly < 1.00.

a AMI is a subset of circulatory diseases.

b CI does not include 1.00.

c HR not calculable because of zero cells.
